# Neuronal Damage and Neuroinflammation, a Bridge Between Bacterial Meningitis and Neurodegenerative Diseases

**DOI:** 10.3389/fncel.2021.680858

**Published:** 2021-06-03

**Authors:** Kristine Farmen, Miguel Tofiño-Vian, Federico Iovino

**Affiliations:** Department of Neuroscience, Karolinska Institutet Biomedicum, Stockholm, Sweden

**Keywords:** bacterial infection, neuronal damage, meningitis, Streptococcus pneumoniae, dementia

## Abstract

Bacterial meningitis is an inflammation of the meninges which covers and protects the brain and the spinal cord. Such inflammation is mostly caused by blood-borne bacteria that cross the blood-brain barrier (BBB) and finally invade the brain parenchyma. Pathogens such as *Streptococcus pneumoniae*, *Neisseria meningitidis*, and *Haemophilus influenzae* are the main etiological causes of bacterial meningitis. After trafficking across the BBB, bacterial pathogens in the brain interact with neurons, the fundamental units of Central Nervous System, and other types of glial cells. Although the specific molecular mechanism behind the interaction between such pathogens with neurons is still under investigation, it is clear that bacterial interaction with neurons and neuroinflammatory responses within the brain leads to neuronal cell death. Furthermore, clinical studies have shown indications of meningitis-caused dementia; and a variety of neurodegenerative diseases such as Alzheimer’s disease, Parkinson’s disease and Huntington’s disease are characterized by the loss of neurons, which, unlike many other eukaryotic cells, once dead or damaged, they are seldom replaced. The aim of this review article is to provide an overview of the knowledge on how bacterial pathogens in the brain damage neurons through direct and indirect interactions, and how the neuronal damage caused by bacterial pathogen can, in the long-term, influence the onset of neurodegenerative disorders.

## Introduction

The incidence of neurological and neurodegenerative diseases has continuously increased worldwide in the last decades, with an expected rise in the coming years due to the aging of the world population. Dementia, which currently affects more than 50 million people globally, is expected to expand its incidence to over 135 million by 2050 (McManus and Heneka, 2017). However, even though genetic and/or environmental factors have been described in many of such diseases, direct causality has not been clearly established: several genetic mutations are associated with dementia, but the reasons why the pathogenesis occurs, when, and how it does, remain unclear (Patrick et al., 2019). In this regard, the interplay between neurological damage, dementia and pathogenic infections has been increasingly assessed during the last years. Indeed, infectious disease burden seems correlated with neurological damage and neurodegenerative progression (Strandberg et al., 2004; Katan et al., 2013). Bacterial meningitis, the inflammation of the meninges caused by infection of the brain parenchymal tissue due to several infectious agents, remains among the leading infectious diseases worldwide (Van De Beek et al., 2016). *Streptococcus pneumoniae* (the pneumococcus) and *Neisseria meningitidis* (the meningococcus) are the main causes of acute bacterial meningitis in Europe and the USA. Depending on the geographical region, mortality rates range between 20–51% and 3–10% respectively, and up to 50% of survivors present long-term neuronal sequelae, including cognitive impairments and hearing loss (Lucas et al., 2016). *Haemophilus influenzae* type b was the leading cause of bacterial meningitis worldwide before the introduction of vaccination; due to lack of vaccination in developing countries, it is still an important cause of meningitis in these regions (Wahl et al., 2018). On the other hand, the introduction of vaccination programs for certain serotypes of meningococci and pneumococci has dropped the incidence of bacterial meningitis in recent years. At the same time, bacterial meningitis due to serotypes that are not included in the vaccine is increasing (McIntyre et al., 2012). Furthermore, the case fatality rate remains high and the clinical outcomes are highly dependent on good health care systems (Swartz, 2004; Thigpen et al., 2011).

In this mini-review article, we will focus on how the three main etiological causes of bacterial meningitis induce both direct and indirect neuronal damage and promote neuroinflammation. Finally, we will show its burden on the population, in terms of neurological disorders and increased risk of dementia, as well as the current efforts and strategies to prevent brain damage and, ultimately, reduce the risk of neurodegenerative diseases.

## Neuronal Damage in Bacterial Meningitis

The bacterial colonization of the nasopharynx is usually an asymptomatic event (Aniansson et al., 1992; Mook-Kanamori et al., 2011); however, the bacteria can penetrate the mucosal epithelium and basal membrane causing invasive disease (Leib and Täuber, 1999). Meningitis develops if the bacteria enter the systemic circulation, penetrate the blood-brain barrier (BBB), and infect the brain, causing inflammation of the parenchyma and meninges (Iovino et al., 2016). Neuroinflammation may promote neuronal damage, which might have an unrepairable effect on neuronal circuits due to the post-mitotic state of neurons (Herrup and Yang, 2007).

### Direct Damage Caused by *S. pneumoniae* Infection

Among the bacterial effectors responsible for neuronal damage, the cytotoxin of *S. pneumoniae* pneumolysin (Ply) is one of the best characterized. Ply is a 53 kDa protein expressed by the majority of clinically-isolated *S. pneumoniae* and exhibits both cytolytic and immunomodulatory effects (Kalin et al., 1987). Upon release from the bacterium, Ply subunits interact in a cholesterol-dependent manner with the cell membrane causing the generation of a pore ~300 Å in diameter, which is cytotoxic to the cell (Mitchell and Dalziel, 2014). In patients suffering from pneumococcal meningitis, Ply was detected in the cerebrospinal fluid (CSF). Furthermore, non-surviving patients had increased Ply levels in the CSF 48 h after hospitalization compared to survivors, indicating the potential deleterious role of this protein on mortality (Wall et al., 2012). Although one early report on the role of Ply in meningitis affirmed that rabbits infected with a Ply deficient *S. pneumoniae* strain showed similar meningeal inflammation pathogenesis compared with rabbits infected with wild-type *S. pneumoniae* strain (Friedland et al., 1995), several other studies disagree with these results. In guinea pigs inoculated with *S. pneumoniae*, Ply was shown to cause cochlear damage and consequently hearing impairment (Winter et al., 1997). Additionally, both *in vitro* and *in vivo* studies affirm the cytotoxicity of Ply, as exposure of Ply to neurons caused cellular damage; and Ply deficiency, reduced virulence of the bacteria (Braun et al., 2002, 2007; Wellmer et al., 2002; Robert et al., 2008; Reiß et al., 2011). The mechanism behind the Ply-induced neuronal death *in vitro* has been shown to be due to, at least in part, the increased intracellular levels of Ca^2+^, resulting in a disruption of the mitochondrial function and activation of apoptosis-induced factors (Braun et al., 2002; Stringaris et al., 2002).

Ply also mediates indirect pathological effects on brain fitness (Braun et al., 2002; Stringaris et al., 2002). First, it damages ciliary ependymal cells in the ventricles, which then reduces the ciliary beating frequency (Mohammed et al., 1999; Hirst et al., 2000). Fully functional ciliary beating is crucial for controlling the CSF volume, transportation of macromolecules, and removal of waste (Olstad et al., 2019). Thus, non-functional ciliary cells most likely contribute to the neuropathological effects in pneumococcal meningitis. Second, it interacts with immune cells in a toll-like receptors 4 (TLR 4)-independent fashion, promoting the release of pro-inflammatory cytokines (McNeela et al., 2010); and third, it induces astrocytic shrinkage, impairing synaptic functionality but also mediating easier spread of bacteria and toxins in brain regions (Förtsch et al., 2011; Hupp et al., 2012).

It was recently shown that Ply might facilitate the internalization of the pneumococcus into neurons together with the pilus-1, a protein complex with adhesin activity, exposed outside the cell wall, which has been associated with the capacity of pneumococci to interact with and invade different types of host cells (Iovino et al., 2020). More specifically, both the pilus-1 component RrgA and Ply interact with β-actin exposed on the neuronal plasma membrane. This interaction caused disruption of the β-actin filaments with consequent neuronal cell death; an intact actin cytoskeleton was previously reported to inhibit the activation of Ca^2+^ influx, the finding of Ply and RrgA enhancing intracellular Ca^2+^ levels in neurons was likely due to the disruption of β-actin filaments (Rosado and Sage, 2000; Tabusi et al., 2021). In the case of RrgA, the, to this date, unknown mechanism behind this process may involve its D3-domain, which has been shown to exhibit an integrin-like fold and may directly interact with β-actin filaments, altering their structure. Furthermore, the co-localization of β-actin and the pneumococcus, even after internalization, suggests that the bacteria use the neuronal cytoskeleton to become internalized (Tabusi et al., 2021).

Additionally, the reactive oxidative species hydrogen peroxide (H_2_O_2_) is produced directly by the pneumococcus, and through its secretion causes DNA damage and epithelial cell death (Spellerberg et al., 1996; Rai et al., 2015). Indeed, primary murine neurons suffered increased apoptosis when treating them with H_2_O_2_, through inhibition of mechanistic target of rapamycin (mTOR) signaling (Chen et al., 2010). H_2_O_2_ also caused microglia apoptosis, possibly synergically together with Ply (Braun et al., 2002). In human brain endothelial cells (hBMECs), Ply and H_2_O_2_ caused apoptosis independently of TLR2 and TLR4 signaling (Bermpohl et al., 2005).

*N. meningitidis* is also known to produce direct damage and cell death to several cell types, but to our knowledge, no studies have reported direct damage to neurons. Two of the most important virulence factors in *N. meningitidis* are PorB and type IV pilus. While the pilus mediates attachment to the plasma membrane, both have been shown to trigger an influx of Ca^2+^ in epithelial cells; in the case of PorB, this has been directly linked with apoptosis (Müller et al., 1999; Tzeng and Stephens, 2000). Due to the relevance of Ca^2+^ concentration in cells and, as these processes are evolutionary conserved, we can hypothesise that they may also mediate neuronal damage. However, direct experimental data on neurons is required to confirm it.

### Indirect Neuronal Damage: Neuroinflammation in Bacterial Meningitis

In several neurodegenerative diseases, including dementia, chronic neuroinflammation is associated with the disease and is also importantly observed prior to neuronal degeneration (Frank-Cannon et al., 2009). While, in the initial phases of the disease, neuroinflammation and the following clearance of unwanted pathogens or non-degradable proteins is desirable, it also mediates harmful effects on the brain environment both in the short and long-term. Neuroinflammation causes the release of several cytotoxic compounds, including reactive oxidative species and nitric oxide, which can stimulate the release of pro-apoptotic compounds, ultimately leading to apoptosis of neurons and other brain resident cells (Lyman et al., 2014). Because neurons are in a post-mitotic state, this has potential deleterious effects as it contributes to neuronal degradation without future replacement of cells (Herrup and Yang, 2007). It is known that bacterial meningitis-induced neuroinflammation causes neuronal degradation (Kim, 2003). This has the potential to be an increased risk factor for the development of neurological diseases, including dementia.

The major players of neuroinflammation are the microglia, the macrophages of the brain, and infiltrating peripheral immune cells (Becher et al., 2017). The use of immunosuppressants in therapy against meningitis has proven to be beneficial for patients, indicating that the pro-inflammatory response itself mediates some of the most pathological effects in the brain (De Gans and van de Beek, 2002). The bacterial invasion of the brain begins with the traversal through the protective BBB, which is composed of brain microvascular endothelial cells, astrocytes, and pericytes, and regulates the movement of active agents, both molecules and cells, in and out the brain (Kim, 2008). Pathogens cross the BBB by three main mechanisms: transcellular migration, para-cellular migration, and internalized in macrophages in a “trojan horse” way (Barichello et al., 2013). The high mortality rate in meningitis patients (even after the introduction of antibiotic treatment) has been linked to inefficiency in the clearance of the bacteria from the brain and the infiltration of peripheral immune cells that cause increased cranial pressure (Van De Beek et al., 2002; Liechti et al., 2015). The microglial cells become activated in response to the bacterial presence and induce a phagocytic response in order to clear the infection. Additionally, bacterial components are recognized, causing an inflammatory response and release of chemo and cytokines. In synergy with chemoattractants produced by other brain resident cells, this causes the infiltration of peripheral immune cells (Barichello et al., 2016). On hospitalization, bacterial meningitis patients present a leaky BBB, a feature observed in other neurological diseases that is known to be related to the infiltration of peripheral immune cells and contributes to the neuroinflammation (Sharief et al., 1992; Stolp and Dziegielewska, 2009). Although lack of neutrophil infiltration was shown to cause more severe disease in experimental bacterial meningitis, this was attributed to the reduced clearance of the bacteria from the brain (Aust et al., 2018). This points out the importance of the balance between beneficial and deleterious neuroinflammation. While studies on the role of peripheral immune cells in bacterial meningitis is scarce, the role of microglia has been thoroughly reviewed elsewhere (Barichello et al., 2016; Thorsdottir et al., 2019). In this review article, we will focus on the mechanism behind the innate immune response to the respective bacteria.

Among biological systems for immune response, TLRs are known to recognize bacterial compounds such as lipoteichoic acid, peptidoglycans, and lipopolysaccharides, but also other bacterial components can interact with these receptors (Schröder et al., 2003). While different TLRs expression varies between brain cells, most of them use the same intracellular adapter protein, myeloid differentiation factor 88 (Myd88), as a transducer (Takeda et al., 2003; Kielian, 2009). This factor interacts with receptor-associated kinase-4, which in turn mediates the activation of the tumor necrosis factor (TNF) receptor-associated factor family, the translocation of nuclear factor (NF)-κB to the nucleus, and the activation of a wide range of genes implicated in the elicitation of the immune response, both in terms of lymphocyte activation and in the production of cytokines and chemokines (Kawasaki and Kawai, 2014). This mechanism has been observed in innate immune cells, including microglia, but also in other brain resident cells (Kopitar-Jerala, 2015). Lymphocytes, on the other hand, have been shown to infiltrate the brain tissues, thus contributing to the neuroinflammatory response (Hoffmann et al., 2015).

Stimulation of peripheral blood mononuclear cells (PBMCs) with *S. pneumoniae, N. meningitidis*, or *H. influenzae* caused a significantly increased expression of NF-κB and the cytokines interleukin (IL)-6, IL-8, and TNF-α compared to untreated PBMCs. Interestingly, *N. meningitidis* induced the highest TNF-α expression (Mogensen, 2006)*. H. influenzae* has been shown to interact with both TLR2 and TLR4 (Wang et al., 2002; Galdiero et al., 2004). Porin on the outer membrane of *H. influenzae* type B activated monocytes by interacting with TLR2, dependently on the co-expression of TLR2 and CD14 on the surface, and the downstream signaling protein Myd88 (Galdiero et al., 2004). Furthermore, lipooligosaccharides (LOS) activated TLR4, but LOS with reduced acetylation activated TLR2, indicating that, through slight modification, bacteria can interact with different receptors (Lorenz et al., 2005). *N. meningitidis* LOS is also a major inducer of inflammation and a tenfold reduction in TNF-α levels has been reported in LOS mutant compared to the wild-type strain (Alison et al., 2001). Additionally, capsule polysaccharides have been shown to cause the release of TNF-α, IL-6, IL-8, and CXCL10, mediated through TLR2 and TLR4/MD-2 pathways (Zughaier, 2011). Meningothelial cells release cytokines when in contact with the meningococcus, with IL-6, CXCL10, and CCL5 levels reduced by up to 90% in TLR4 knockouts (Royer et al., 2013). PorB binds to TLR2/TLR1 causing increased activation of NF-κB as measured by IL-8 induced levels (Massari et al., 2006). Finally, *S. pneumoniae* Ply has been shown to induce inflammation in a TLR4 dependent, but also independent pathway, while lipoteichoic acid interacts with the TLR2 (Malley et al., 2003; Schröder et al., 2003; McNeela et al., 2010). Furthermore, TLR1/2 levels were increased upon *S. pneumoniae* stimulation, and knockout of TLR9 caused mice to be more susceptible to disease; implementing also these TLR receptors in the innate immune sensing of *S. pneumoniae* (Schmeck et al., 2006; Albiger et al., 2007).

Bacterial compounds also elicit a cellular response through intracellular mechanisms. In this regard, NOD-like receptors are intracellular receptors whose major downstream activating pathways are the NF-κB and the mitogen-activated protein kinase (MAPK) pathways, resulting in increased pro-inflammatory cytokine production (Chen et al., 2009). Peptidoglycans expressed on the bacterial cell surface are recognized by the intercellular receptor NOD2 (Sorbara and Philpott, 2011). In mpneumococcal-induced meningitis, both microglia and astrocyte recognize *S. pneumoniae* components through the NOD2 receptor, causing increased nuclear translocation of NF-κB and release of IL-6 and TNF-α from both cell types. Furthermore, this has been associated with elevated astrogliosis and demyelination in the corpus callosum (Liu et al., 2010). Interestingly, this has also been reported in *N. meningitidis*-induced meningitis (Chauhan et al., 2009). Clearly, this indicates the central role of the NOD2 receptor in the contribution to a deleterious inflammation response that results in neuronal damage in bacterial meningitis. Monocytes treated with LOS also show increased NOD2 expression (Choi et al., 2014). Peptidoglycans released by meningococci are detected by NOD1 and induce an inflammatory response to these (Woodhams et al., 2013). Taken together these results provide evidence for the role of NOD-like receptors in the meningitis neuroinflammatory state. Three NOD-like receptor protein families (NLRP1, NLRP3, and NLRC4) can assemble to generate an inflammasome, leading to caspase-1 activation and cleaving of the precursors of IL-1β and IL-18 into their active counterparts (Gross et al., 2011). NLRP3 has been shown to be an important factor in the pathology of meningitis (Hoegen et al., 2011) In pneumococcal-induced meningitis, the production of interferon (IFN)-γ was dependent on the protein ASC, which is an adaptor protein for multiple inflammasomes (Mitchell et al., 2012). Ply is also able to induce the expression of the inflammasome, independently of TLR4 interaction (McNeela et al., 2010; Hoegen et al., 2011).

**Figure 1:**
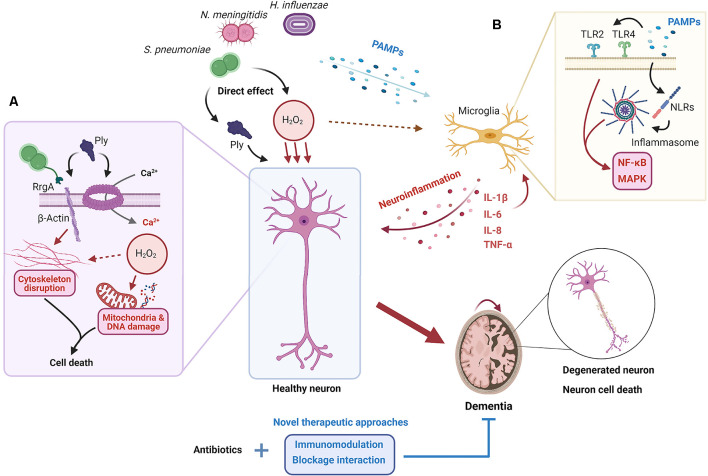
Proposed mechanisms for neuronal damage during bacterial meningitis. When the pathogens *S. pneumoniae*, *N. meningitidis*, and *H. influenzae* invade the brain, both the action of their virulence factors and the elicited neuroinflammatory response cause neuronal damage and death. **(A)** Pores in the neuron plasma membrane formed by pneumolysin (Ply), released by *S. pneumoniae*, result in the influx of extracellular Ca^2+^. At the same time, the interaction between β-actin, exposed outside the plasma membrane, with Ply and RrgA causes the disruption of the actin cytoskeleton. This, coupled with the release of H_2_O_2_, oxidative outburst, and subsequent mitochondrial and DNA damage, results in neuronal death. **(B)** Several pathogen-associated molecular patterns (PAMPs) are recognized by microglia through receptors such as TLRs and NLRs, which results in the activation of nuclear factor (NF)-κB and the mitogen-activated protein kinase (MAPK) pathway and the release of pro-inflammatory cytokines and other mediators, and the recruitment of inflammatory cells. The establishment of a neuroinflammatory state inside the brain leads to neuronal death through different mechanisms such as tumor necrosis factor (TNF)-α overproduction or oxidative outburst. Ultimately, neuronal death results in neurological sequelae and potential long-term dementia; something that may be prevented if current antibiotic treatments are coupled with new therapeutic approaches based on immunomodulation and/or blockage of direct interaction between bacteria and cells.

In the extracellular milieu, cytokines modulate and regulate the inflammatory response on target cells and are important for the clearance of unwanted products, but also for inhibition of excessive immune responses (Kany et al., 2019). In bacterial meningitis, the cytokines released are dependent on the stimuli, signaling receptor and cell type. It has been shown that IL-1β and IL-18 levels are upregulated during bacterial meningitis and, specifically, IL-1β levels are correlated with leukocyte levels in the CSF and neuronal sequelae in patients (Mustafa et al., 1989; Fassbender et al., 1999). TNF-α expression has been shown to be upregulated in the brain after pneumococcal-induced meningitis (Barichello et al., 2009). When comparing meningococcal-induced bacteremia and meningitis in patients, TNF-α levels were significantly higher in the latter (Waage et al., 1989). Microglia, astrocytes and neurons express TNF-α receptors (TNFR), with TNFR1 being the most abundant (Barichello et al., 2009). Through this receptor, TNF-α can induce apoptosis of cells in already stressed cells; therefore, an adjuvant therapy against TNF-α could be relevant for treatment against bacterial meningitis, and indeed this has been shown to attenuate neuronal death in rats (Leib, 2001; Bhardwaj and Aggarwal, 2003). hBMECs have increased IL-8 and IL-6 production compared to peripheral endothelial cells when exposed to *N. meningitidis*. These cytokines are important for the activation of the immune system and indicate that the hBMECs themselves contribute to the increased pro-inflammatory milieu in the brain (Dick et al., 2017). Both direct and indirect mechanisms of neuronal damage in bacterial meningitis have been summarized in Figure 1.

## Infection Burden and The Epidemiology of Dementia

As stated in the introduction, the relevance of the infectious etiology has been increasingly stressed in several neurodegenerative diseases, such as Alzheimer’s disease (AD; Sochocka et al., 2017), Parkinson’s disease (PD; Brudek, 2019), and Rapidly Progressive Dementia (Geschwind, 2016). However, a clear relationship between dementia and infectious alterations of normal physiology is difficult to establish for several reasons. Sepsis, on the other hand, a grave condition which can be caused by several pathogens, is known to produce severe BBB dysfunction (Barichello et al., 2021), microglial activation (Li et al., 2020), acute neuroinflammation, brain injury and cerebral dysfunction (Meneses et al., 2019; Gu et al., 2021), and long-term cognitive and functional impairments (Brown, 2019; Rengel et al., 2019). This is also true for bacterial meningitis, especially in the case of neonates (Heath et al., 2011) and young infants (Hsu et al., 2018).

In patients with AD both the onset and the progression of the disease has been associated with a history of infection in the patient’s life; in particular, the incidence of pneumonia, as well as respiratory and urinary tracts infections, has been shown to be higher in AD patients, traditionally considered a consequence of the disease, but can also be related to its onset (Kountouras et al., 2006; Natalwala et al., 2008; Miklossy, 2011; Too et al., 2021). Delirium, on the other hand, often caused by CNS infection, is correlated with an acceleration in the progression towards dementia (McManus and Heneka, 2017). Dunn and colleagues also found an association between dementia and infectious disease in a case-control study (Dunn et al., 2005). Bacterial periodontitis, a common ailment in the elderly, has also been shown to correlate with cognitive decline and AD (Ide et al., 2016).

Eradication of *Helicobacter pylori* infection has been hinted to be beneficial in hampering AD progression (Kountouras et al., 2009). Leprosy has also been linked to dementia (Su et al., 2012), though anti-leprosy drugs do not appear to have an effect in the prevention of AD neurotoxicity (Endoh et al., 1999).

This relationship between the CNS and microbiological agents does not restrict itself to infectious pathogens, however. The gut microbiota now seems to play an important homeostatic role in the brain, as shown both in human (Paley, 2019) and mice, in which the Apolipoprotein E genotype —the strongest prevalent risk factor for AD development— has been sharply associated with specific gut microbiome profiles (Tran et al., 2019). This has led to the definition of a brain-gut-microbiota axis in which d-glutamate metabolized by the gut may significantly contribute to or hamper the progression of AD (Chang et al., 2020). Also, a 12-week supplementation of *Bifidobacterium breve* A1 has shown a promising effect in preserving cognitive function in elderly subjects with memory loss complaints (Kobayashi et al., 2019).

Evidence of the relationship between infectious burden and neurodegeneration is not restricted to AD. A case-control study by Vlajinac and colleagues showed a correlation between PD and several infectious agents such as *H. pylori* (Bjarnason et al., 2005; Shen et al., 2017; Dardiotis et al., 2018). *H. pylori* infection has also been associated with multiple sclerosis (MS), as a putative protective factor (Jaruvongvanich et al., 2016; Yao et al., 2016). In MS, an infection by *Chlamydia pneumoniae* is considered a risk factor (McKay et al., 2015). Lastly and interestingly, the risk for schizophrenia is enhanced in offspring exposed to microbiological infections (Brown and Susser, 2002), and in bacterial-infected pregnant women’s offspring (Sørensen et al., 2008).

Bacterial meningitis is well known to potentially produce neuronal damage even after pathogen clearance and good prognosis. In 2002, Van de Beek and colleagues reported the presence of long-term cognitive sequelae in patients who had recovered well after pneumococcal meningitis; 27% presented significative cognitive slowness (Van De Beek et al., 2002). Indeed, a later study showed similar cognitive disabilities in patients with moderate disability after bacterial meningitis than patients with good recovery, hinting at a similar risk for further long-term neurological damage (Weisfelt et al., 2006). Later reports have confirmed the neuropsychological sequelae of bacterial meningitis, specifically cognitive slowness, epilepsy, and hearing loss, but also affected learning and memory functions, poorer performance in executive functions, language, and verbal tests (Schmidt et al., 2006; Hoogman et al., 2007; Christie et al., 2017). The risk of at least one major sequelae (cognitive deficit, bilateral hearing loss, motor deficit, seizures, visual impairment, hydrocephalus) has been estimated at 13%, this percentage rises to almost 25% in pneumococcal meningitis (Grimwood et al., 1995; Edmond et al., 2010). Neonatal pneumococcal meningitis leads to cognitive impairment in 30–52% of surviving patients (Barichello et al., 2013). Furthermore, *S. pneumoniae*-induced meningitis in childhood and adolescence has been linked with long-term neurological damage. After ≥14 years of pneumococcal meningitis diagnosis and treatment, patients showed significantly lower full scale and verbal IQ, numeracy or school functioning; 14% of them presented partial or profound hearing impairment (Christie et al., 2011). Severe bacterial meningitis, which can cause cerebral infarction, cerebritis, subdural empyema, cerebral abscess or intracerebral bleeding, can lead to grave neurological sequelae such as short-term (Naito et al., 2010) or even long-term cognitive impairment (Singhi et al., 2007; Lucas et al., 2016), epilepsy, and dementia (Kamei, 2016), with critically worse prognosis in the case of neonatal bacterial meningitis (Baud and Aujard, 2013). This epidemiological picture correlates with brain injury observed in bacterial meningitis patients. Vasculitis, intravascular coagulation, and reduced blood flow cause ischemic, necrotic brain injury in the cortex; at the same time, an apoptotic burst has been described in the dentate gyrus of the hippocampus, as a result of a process which involves multiple effects induced by bacteria, their components, and the host immune response (Liechti et al., 2015).

## Translational Approaches to Prevent Infection-Derived Neuronal Damage

By means of direct interaction with cells and/or neuroinflammation, infections in the brain have the potential to provoke neurological sequelae, which may, in the long-term, develop into dementia. Thus, clearance of the infection is not enough to provide a full recovery to patients suffering from different kind of illnesses; novel therapeutic approaches that can either prevent bacterial invasion of the brain, or block bacterial interaction with brain cells, especially neurons, must be developed to reduce the chances of dementia onset in the elderly. To begin with, it is important to clearly identify the bacterial virulence factors that promote brain invasion, activation of the immune system, and neuron cell death.

In pneumococcal meningitis, for instance, polymeric immunoglobulin receptor (pIgR) and platelet endothelial cell adhesion molecule (PECAM-1) has been identified as the receptors on the BBB endothelial cells that mediate invasion of *S. pneumoniae*into the brain (Iovino et al., 2016); a combination of anti-pIgR and PECAM-1 antibodies with β-lactam antibiotics have proven to minimize pneumococci invasion of the brain, a proof of concept of a successful blockade of host-pathogen interaction *in vivo* (Iovino et al., 2017, 2018). However, the infection itself usually disrupts the integrity of the BBB, an advantage for antibiotics to reach the brain but a problem if the approach is to block pathogens crossing the barrier, as most therapies will aim at the aftermath of an infection (Al-Obaidi and Desa, 2018). An alternative may consist in the development of so-called smart carriers, such as bioengineered extracellular vesicles (Saint-Pol et al., 2020; Shahjin et al., 2020), modified liposomes (Zhang et al., 2019) or synthetic nanoparticles (Zhou et al., 2018).

With or without an efficient brain delivery device, biological processes must be correctly targeted to prevent neuronal damage. Among promising findings, brain-derived neurotrophic factor and melatonin were thought to protect against brain injury, improve hearing, and reduce neuronal death in pediatric bacterial meningitis (Grandgirard and Leib, 2006). In terms of modulating the neuroinflammatory process, the anti-inflammatory and immunosuppressive effects of corticosteroids have been well known for decades. In a meta-analysis, corticosteroid administration in bacterial meningitis patients has been shown to prevent hearing loss and short-term neurological sequelae in high-income countries. This effect, however, seems strain-specific and is not observed in low-income countries (Brouwer et al., 2015). Reducing the neutrophil recruitment to the brain, on the other hand, through modulation of apoptosis may be a potential new way to reduce neuronal damage (Principi and Esposito, 2020). Antibiotics themselves contribute to the inflammatory response if they have a bacteriolytic activity, which releases highly inflammatory lysis products (Kietzman and Tuomanen, 2019). Using bactericidal but non-bacteriolytic antibiotics such as daptomycin may bypass this problem (Principi and Esposito, 2020). The adjunction of daptomycin in the treatment of bacterial meningitis has recently proven its therapeutic potential *in vitro* (Maldiney et al., 2021) and it is the subject of an ongoing clinical trial to improve the prognosis and survival of pneumococcal meningitis (AddaMAP, NCT03480191). Finally, due to its relevance in neuroinflammation and brain disease, and its implication in neuron cell death, inflammasome modulation is considered a promising target in the context of bacterial meningitis (Walsh et al., 2014) and neurodegenerative diseases (Heneka et al., 2018).

Ultimately, targeting the direct interaction between pathogenic agents and neurons maybe a third alternative, though many of the molecular mechanisms involved in such interactions are focused on immune cells or the BBB, poorly characterized, or not known. In *S. pneumoniae* bacterial meningitis, we have recently shown that the bacteria attachment and invasion of the neuron is mediated through RrgA and Ply interaction with exposed β-actin on the plasma membrane (Tabusi et al., 2021). Preventing or blocking altogether this kind of direct interactions between neurons and infectious agents may well provide novel translational approaches to prevent brain damage and dementia.

## Concluding Remarks

While the deleterious effects of bacterial infections on neurological function are now clear in several models of disease, the sequelae of such infections, from hearing loss to motor and cognitive dysfunctions remain highly prevalent. In pneumococcal-induced meningitis, more systematic and epidemiological studies are required in order to assess the importance of different virulent factors such as RrgA or Ply, whose mechanisms of action in neurons could explain much of the short-term and long-term neuronal damage observed in recovered patients (Tabusi et al., 2021). Additionally, long-term epidemiological studies of survivors from bacterial meningitis may clarify the relationship between brain infection and the onset of dementia. Finally, treatments targeting neuroinflammation and neuronal damage may prove useful to prevent the development of neurodegenerative diseases.

## Author Contributions

KF and MT-V wrote the manuscript draft. FI designed the overall theme and provided supervision. All authors contributed to the article and approved the submitted version.

## Conflict of Interest

The authors declare that the research was conducted in the absence of any commercial or financial relationships that could be construed as a potential conflict of interest.
